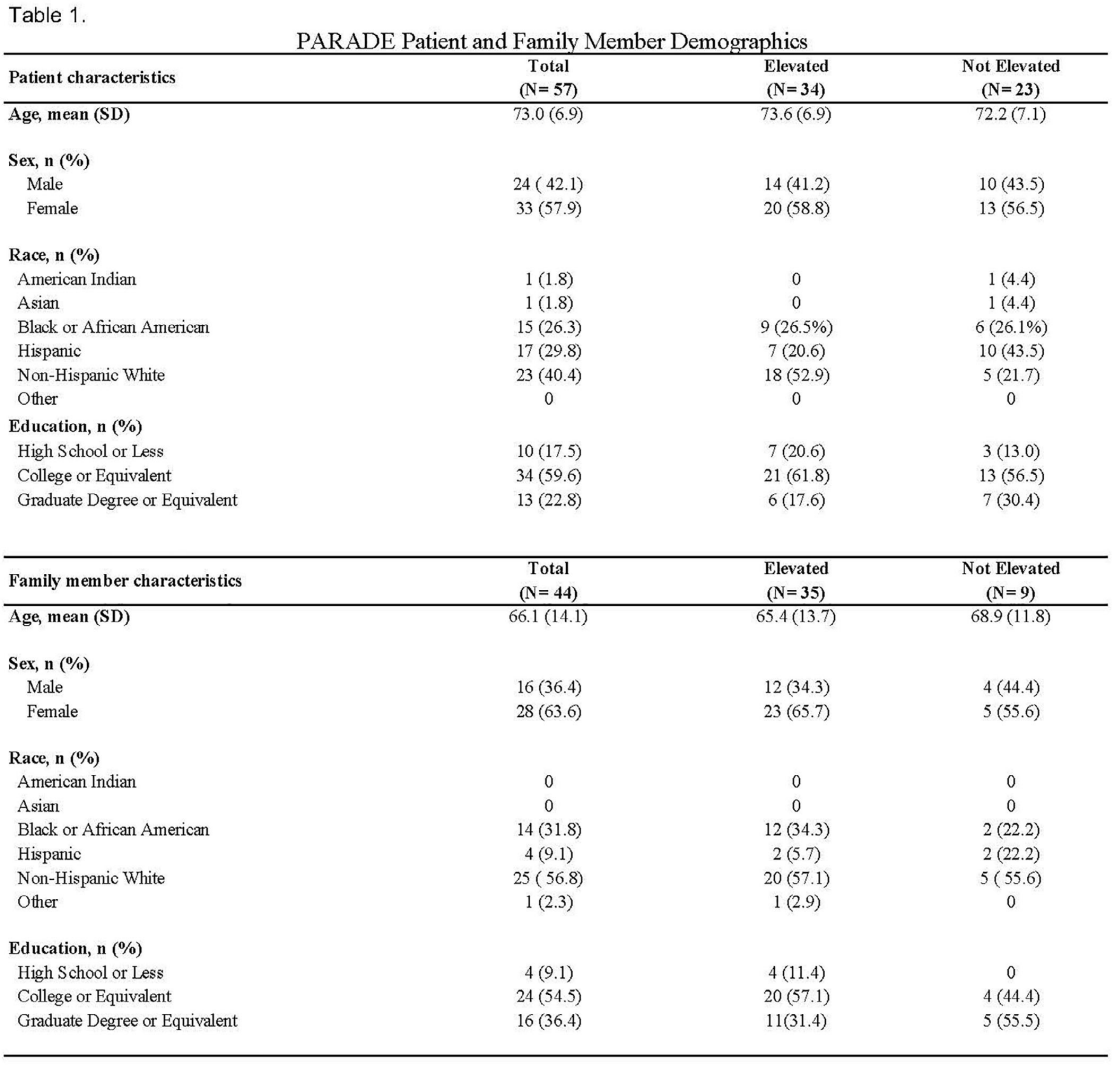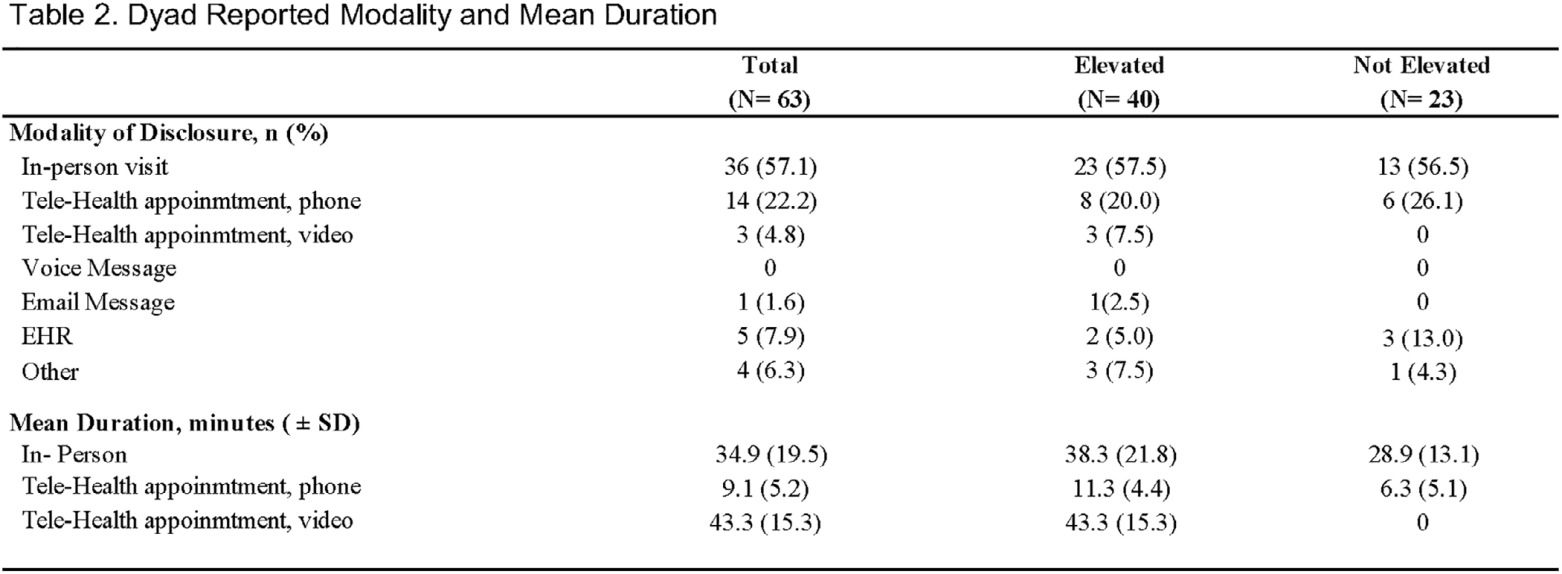# Perceptions of Biomarker Disclosure Experiences Among ADRD Patients and Family Members: the PARADE Study Experience

**DOI:** 10.1002/alz70861_108964

**Published:** 2025-12-23

**Authors:** Melany Medina, Eunji Russ, Megan G Witbracht, Dianxu Ren, Melissa L Knox, Jennifer H Lingler, Josh D Grill

**Affiliations:** ^1^ Institute for Memory Impairments and Neurological Disorders, University of California, Irvine, Irvine, CA USA; ^2^ University of Pittsburgh Alzheimer's Disease Research Center (ADRC), Pittsburgh, PA USA

## Abstract

**Background:**

Most available data on Alzheimer’s Disease and Related Dementias (ADRD) biomarker disclosure have come from well‐controlled research studies. Less is known about real‐world experiences. We assessed ADRD biomarker disclosure experiences among diverse participants in the ongoing Patient And family members Reactions to biomarker‐informed ADRD DiagnosEs (PARADE) study. PARADE began as an approved add‐on study to the CMS Coverage with Evidence Development New IDEAS study of amyloid PET imaging.

**Method:**

Analyzed data were self‐reported and limited to enrolled PARADE participants recruited from the New IDEAS study. Data were collected through interviews 4–6 weeks post‐disclosure. We compared the manner of disclosure of amyloid PET results (e.g., in‐person, phone telehealth, video telehealth, voice message, email message, electronic health record (EHR), and other) and the mean duration of these clinical interactions between those receiving elevated vs. not elevated amyloid results. We used family member‐reported duration data if it was available.

**Result:**

Data from 63 dyads were analyzed (Table 1). In‐person disclosure visits (*n* =36) were most common across biomarker outcomes (Table 2) and similar in frequency for elevated (23/40) compared to not elevated (13/23) amyloid results. After in‐person visits, phone telehealth (*n* =14) was the next most frequent manner of disclosure. Six individuals learned their results through email or the electronic health record. In‐person visits (mean±SD, 34.9±19.5) were longer than phone telehealth visits (9.1±5.2) and this was consistent for those with elevated and not elevated amyloid (Table 2). Visits for individuals with elevated compared to not elevated amyloid were longer for in‐person visits (38.3±21.8 vs 28.9±13.1) and phone telehealth visits (11.3±4.4 vs 6.3±5.1).

**Conclusion:**

These are among the first real‐world data pertaining to biomarker‐informed diagnostic disclosure of cognitive impairment. The data suggest clinicians may spend longer explaining elevated compared to not elevated amyloid results. Participants with telehealth phone visits reported substantially shorter interactions with the clinician, compared to those seen in‐person. Future research should examine how contextual factors influence disclosure experiences.